# Knockdown Resistance (*kdr*) Mutations I1532T and F1534S Were Identified in *Aedes albopictus* Field Populations in Zhejiang Province, Central China

**DOI:** 10.3389/fcimb.2021.702081

**Published:** 2021-06-29

**Authors:** Yuyan Wu, Qinmei Liu, Yunpeng Qi, Yinping Wu, Qinxiang Ni, Weihua Chen, Jinna Wang, Tianqi Li, Mingyu Luo, Juan Hou, Zhenyu Gong, Jimin Sun

**Affiliations:** ^1^ Department of Infectious Diseases Control and Prevention, Zhejiang Provincial Center for Disease Control and Prevention, Hangzhou City, China; ^2^ Department of Infectious Diseases Control and Prevention, Jiaxing Center for Disease Control and Prevention, Jiaxing City, China; ^3^ Department of Vector Control and Prevention, Yiwu Center for Disease Control and Prevention, Yiwu City, China; ^4^ Department of Infectious Diseases Control and Prevention, Wenzhou Center for Disease Control and Prevention, Wenzhou City, China; ^5^ Department of Infectious Diseases Control and Prevention, Quzhou Center for Disease Control and Prevention, Quzhou City, China

**Keywords:** *Aedes albopictus*, pyrethroid, insecticide resistance, *kdr*, Central China

## Abstract

*Aedes albopictus* is the only vector that can transmit the dengue virus in Zhejiang Province, central China, and it can develop insecticide resistance due to long-term exposure to pyrethroids. The presence of knockdown resistance (*kdr*) mutations is one of the mechanisms responsible for pyrethroid resistance, and has been reported in some *Ae. albopictus* populations in southern China. However, little is known about the DNA diversity of the voltage-gated sodium channel (VGSC) gene in *Ae. albopictus* populations in central China. Four *Ae. albopictus* field populations were collected, in Yiwu (YW), Quzhou (QZ), Wenzhou (WZ), and Jiaxing (JX) from Zhejiang Province, central China. The susceptibility of *Ae. albopictus* adults to three pyrethroids (beta-cypermethrin, deltamethrin, and permethrin) was tested using the WHO tube assay, and *Kdr* mutations were identified *via* PCR and sequencing. The relationship between *kdr* mutations and pyrethroid phenotypes was also analyzed. Of the four populations, none was sensitive to any pyrethroid tested, and the YW population showed the strongest pyrethroid resistance. Non-synonymous *kdr* mutations were detected in codons 1532 and 1534, domain III. At codon 1534, one mutant allele, TCC(S), was detected in the four populations with a frequency of 42.08%, while at codon 1532, one mutant allele, ACC(T), was detected in the JX and QZ populations, with frequencies of 4.22 and 3.03%, respectively. The F1534S mutant allele was positively correlated with both beta-cypermethrin and deltamethrin resistance phenotypes (OR > 1, P < 0.05), whereas the I1532T mutant allele was possibly negatively correlated with beta-cypermethrin, deltamethrin, and permethrin resistance phenotypes (OR < 1, P > 0.05). In conclusion, resistance and resistance mutations regarding to three pyrethroids are already present in the *Ae. Albopictus* populations from Zhejiang, central China, which prompts the need to use non-insecticide-based methods of insect control.

## Introduction

*Ae. albopictus*, also known as the Asian tiger mosquito, is widely distributed in southern and central China (Robertson and Hu, 1988). It is perhaps the most dangerous mosquito vector species in Zhejiang Province because of its high density and primary role in transmitting Zika, chikungunya, and dengue viruses (Robertson and Hu, 1988; Yang and Fu, 2006). In 2019, 924 cases of dengue fever were reported in Zhejiang Province, China, which was 3.90-fold higher than that in 2018 (237 cases). *Ae. albopictus* is the only vector species responsible for chikungunya and dengue fever in Zhejiang Province and its surrounding areas, which needs to be controlled (Guo et al., 2016).

Elimination of larval breeding sites and insecticide application are the two main means of controlling *Aedes* species. At present, the insecticides have been employed as the principal control procedure because of its excellent quick kill effect when *Aedes*-borne diseases spread (WHO, 2013; Wang and Jiang, 2016; Wang et al., 2017). As a consequence, insecticides such as pyrethroids have been widely used by both government campaigns and citizens. According to our previous research, the consumption of pyrethroids in Hangzhou City, Zhejiang Province is 5,566 kg per liter per year (unpublished data). The long-term and heavy utilization of pyrethroids has resulted in the resistance of many populations of *Ae. albopictus*, which poses a significant challenge for its control when it triggers an outbreak of dengue fever (Hou et al., 2017).

Behavioral resistance, target insensitivity, and metabolic detoxification are related to the mechanisms of *Ae.* resistance (Hemingway and Ranson, 2000). Target sensitivity has been widely studied in *Ae. aegypti* for several decades, although little is known about it in *Ae. albopictus* (Kushwah et al., 2015; Wuliandari et al., 2015; Smith et al., 2018). Since the first report of the F1534C mutant allele in *Ae. albopictus* populations in Singapore in 2011, the F1534S and F1534L mutations were then detected in *Ae. albopictus* in Haikou and Guangdong Province, China (Kasai et al., 2011; Wang et al., 2015; Chen et al., 2016; Xu et al., 2016; Li et al., 2018). A few years later, the I1532T mutant allele was identified in populations of *Ae. albopictus* in Shanghai, and a correlation was suspected between the I1532T mutation and pyrethroid resistance caused by the F1534S mutation (Gao et al., 2018). However, the vast majority of studies on *kdr* mutations in *Ae. albopictus* was concentrated in the southern tropics, while most of the *Ae. albopictus* can be found in the subtropical regions of China, where the status of *kdr* mutations remains unclear. Zhejiang Province, located on the east coast of central China, has a subtropical monsoon climate. Although resistance against pyrethroids has been detected for several years, few studies on *kdr* mutations have been conducted in *Ae. albopictus* populations in Zhejiang.

In the present study, we investigated the insecticide resistance of *Ae. albopictus* populations collected from the north, south, east, and west of Zhejiang Province to three pyrethroids (beta-cypermethrin, deltamethrin, and permethrin), and the corresponding *kdr* mutations in these four populations were examined. The aim was to explore the occurrence, frequency, and distribution of possible *kdr* mutations in *Ae. albopictus* in a subtropical climate in central China, and to explore the relationship between *kdr* mutations and pyrethroid resistance.

## Materials and Methods

### Ethics Statement

No permit was required for this field study, because no endangered or protected species were involved, and mosquito collections at breeding sites were provided by the owners.

### Mosquito Sampling

**Figure 1:**
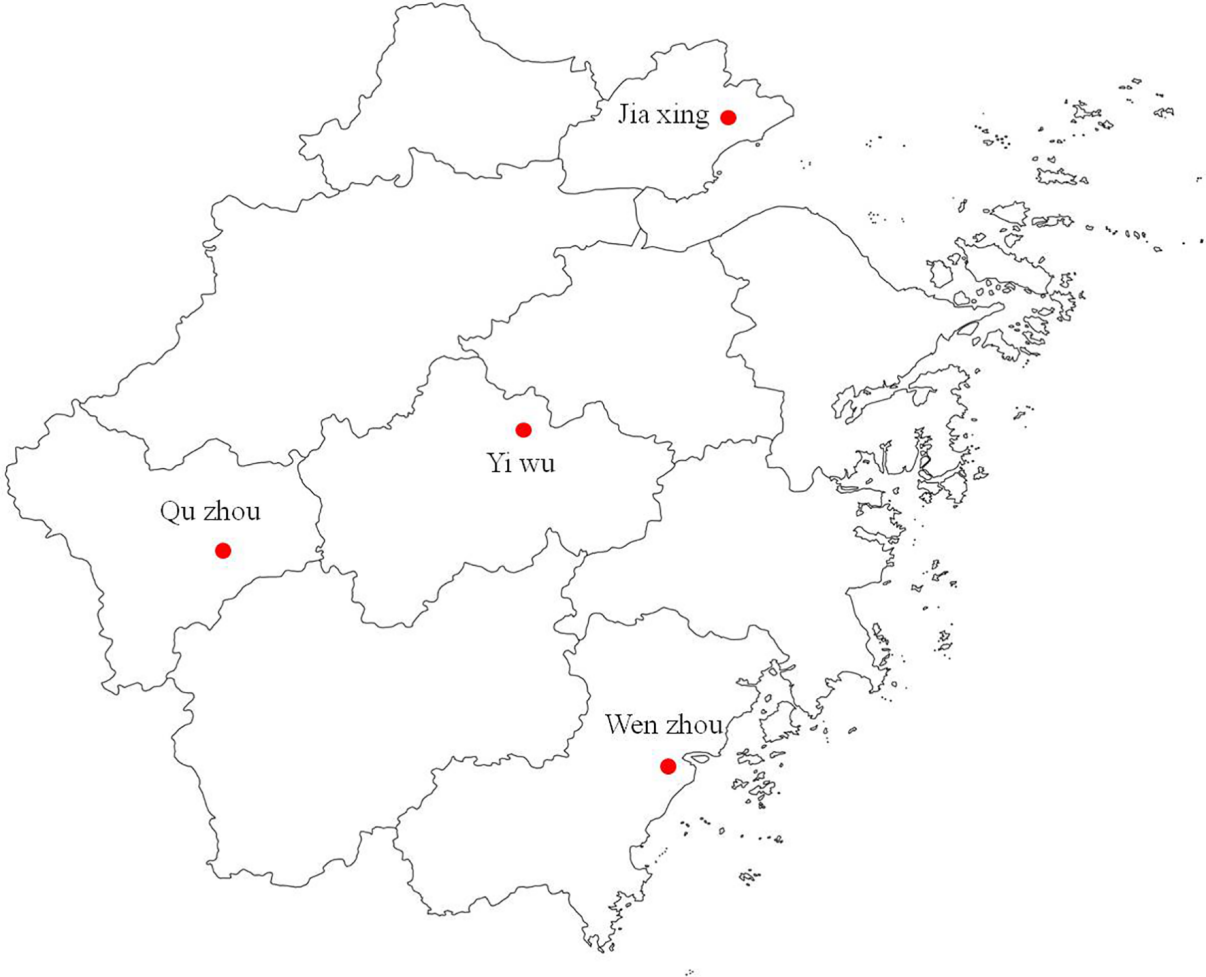
Map of Zhejiang Province showing the *Ae. albopictus* collection sites.

The field populations of *Ae. albopictus* were collected as larvae or pupae from four sites during the 2019 active mosquito season, in Yiwu (YW), Quzhou (QZ), Wenzhou (WZ), and Jiaxing (JX), in Zhejiang Province, China (**Figure 1**). Mosquito larvae were collected from their breeding sites, such as scrap tire dumps, flowerpot trays, plastic containers, and water tanks. All larvae were brought back to the Zhejiang Provincial Center for Disease Control and Prevention Laboratory and were reared in an incubator to adults under standard conditions at (26 ± 1)°C and 65 ± 5% relative humidity with a 14:10 light:darkness period. The F1 progeny adults were used for susceptibility testing.

### Insecticide Susceptibility Bioassay

Non-blood-fed female mosquitoes, 3 to 5 days post-emergence, were tested for susceptibility to three pyrethroid insecticides using WHO tube assay. Test papers containing beta-cypermethrin (0.4%), deltamethrin (0.1%), and permethrin (3%) were used for the assays, which were provided by the National Institute for Communicable Disease Control and Prevention, Chinese Center for Disease Control and Prevention (China CDC) (Chen et al., 2016; Hou et al., 2020). Silicone oil-treated papers without insecticides were used as controls. Tests using each insecticide paper and control paper were repeated at least three times according to the tube test protocol recommended by the China CDC (National Health Commission of the People’s Republic of China, 2016; Hou et al., 2020). After 1 h of exposure, the mosquitoes were transferred to its recovery tube and were maintained in an 8% sucrose solution for 24 h. After 24 h, the mosquitoes were considered alive if they could fly, while they were considered dead if they were knocked down moribund or motionless. The number of dead mosquitoes was counted to calculate the 24-h mortality, and to evaluate insecticide sensitivity. If the control group mortality was between 5 and 20%, the test group mortality would be corrected by Abbott’s formula, as follows: Corrected mortality (%) = (test group mortality − control group mortality)/(1-control group mortality) × 100. If the mortality of the control group was ≥20%, the bioassay would be repeated (National Health Commission of the People’s Republic of China, 2016). Resistance status was classified using corrected mortality according to the WHO recommendations (WHO, 2016). There are three categories according to the corrected 24-h mortality rate: susceptibility, if mortality was between 98 and 100%; probable resistance, if mortality was between 90 and 97%; and resistance, if mortality was <90% (WHO, 2016). All mosquitoes were collected and stored in a −80°C refrigerator for DNA analysis.

### DNA Extraction and *kdr* Allele Detection

Genomic DNA was extracted from individual mosquitoes using the ALLPrepQiagen Nucleic Acid Kit (Qiagen, Germany). Extracted DNA was stored at −20°C until further analysis. To identify *kdr* alleles, partial sequences of domains II, III, and IV of the voltage-gated sodium channel (VGSC) gene, which are known targets of pyrethroid and Dichloro diphenyltrichloroe thane (DDT) insecticides, were amplified using the primers aegSCF3 (5’-GTGGAACTTCACCGACTTCA-3’) and aegSCR22 (5’-TTCACGAACTTGAGCGCGTTG-3’), aegSCF7 (5’-GAGAACTCGCCGATGAACTT-3’) and albSCR9 (5’-CTGATCCTCCGTCATGAACA), and albSCF6 (5’-TCGAGAAGTACTTCGTGTCG-3’) and albSCR8 (5’-AACAGCAGGATCATGCTCTG-3’), respectively (Chen et al., 2016; Gao et al., 2018). The PCR kit was purchased from Aidlab (China). PCR was carried out in Eppendorf AG 22331 Hamburg (Eppendorf, Germany). The cycling parameters used were adapted from the methods of Kasai and Yajun Ma, including one cycle of denaturation at 94°C for 2 min, followed by 35 cycles of amplification at 94°C for 30 s, 60°C for 30 s, and 72°C for 30 s, with a final extension at 72°C for 8 min (Kasai et al., 2011; Gao et al., 2018). After electrophoresis, the PCR products were purified and directly sequenced in both directions using the same set of primers (Gao et al., 2018). Sequences were analyzed using DNASTAR Lasergene 12.0 software to determine the codons and the corresponding genotypes (Burland, 2000).

### Statistical Analysis

The frequency of a particular allele was calculated for each population as the number of alleles/(sample size × 2). Chi-squared tests were used, and the odds ratio (OR) values with 95% confidence intervals (CI) were calculated using SPSS (version 23.0, Armonk, NY: IBM Corp, USA) (Deng et al., 2017) to examine the association between *kdr* alleles and the resistance phenotypes. In this study, the dependent variable was the mosquito status, which was either dead or alive at 24-h post bioassay. Dead mosquitoes were defined as having a susceptible phenotype while live mosquitoes were defined as having a resistant phenotype. If the OR > 1, the relationship between the *kdr* allele and resistant phenotype was considered as positive. If the OR < 1, the relationship between the *kdr* allele and resistant phenotype was considered as negative (Gao et al., 2018). P < 0.05 was considered statistically significant.

## Results

### Insecticide Susceptibility Tests

**Figure 2:**
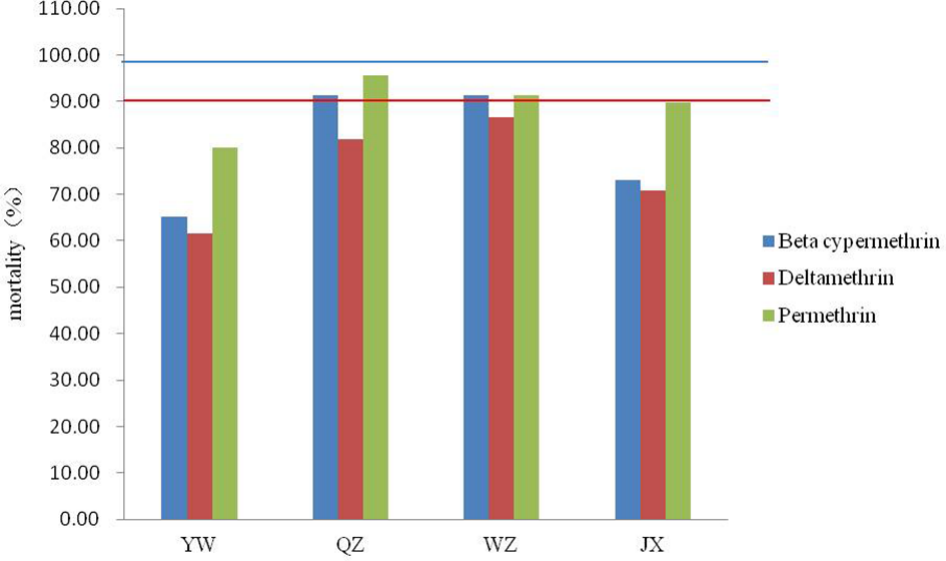
Mortality of four *Ae. albopictus* field populations after exposure to three pyrethroids using the WHO tube bioassay. Red lines represent 90% mortality and blue lines represent 98% mortality.

None of the four field populations of *Ae. albopictus* showed sensitivity to the pyrethroids tested (**Figure 2**). The range of corrected mortality was between 65.15% (YW) and 91.38% (QZ) after exposure to beta-cypermethrin, 61.67% (YW) and 86.67% (WZ) after exposure to deltamethrin, and 80.00% (YW) and 95.52% (QZ) after exposure to permethrin. The resistance of the YW population was the highest among all three pyrethroids tested, while the QZ population was relatively sensitive to beta-cypermethrin and permethrin. Among the three pyrethrin insecticides tested, permethrin caused the highest mortality in all four populations.

### Detection of *kdr* Genes in *Ae. albopictus* Field Populations

One hundred fifty-three specimens were sequenced for domains II and IV of the VGSC gene, and we found synonymous mutations in both domains, although they were not recorded in this study. Sequences of domains III were obtained from a total of 385 samples, and non-synonymous *kdr* mutations were detected at codons 1532 and 1534. At codon 1532, in addition to the wild-type codon ATC encoding isoleucine (I), another allele was detected, which was ACC, encoding threonine (T) (GenBank accession No.: MH384955 − MH384958) (Gao et al., 2018). The mutant allele I1532T was detected in the JX and QZ populations, with frequencies of 4.22 and 3.03%, respectively. Three genotypes, including the wild genotype I/I (91.86%) and the wild/mutant heterozygote I/T (8.14%) in the two populations were detected, but the mutant genotype T/T was not detected in this study.

At codon 1534, only two alleles were detected in samples obtained from the Zhejiang Province: one mutant allele TCC/S and the wildtype TTC/F (**Table 1**). The mutant allele TCC/S was found in all four populations. Overall, the allele frequencies were 57.92 and 42.08% for TTC/F and TCC/S, respectively, were obtained, and a total of three genotypes, including the wild-type genotype F/F (22.86%), wild-type/mutant heterozygote F/S (38.70%), and mutant genotype S/S (38.44%). Among the four populations, the mutant frequency was highest in YW (88.37%) and lowest in JX (42.77%).

**Table 1:** *Kdr* mutant allele frequency at codon 1534 from *Ae. albopictus* populations in YW, QZ, WZ, and JX from Zhejiang Province, China.

Insecticide	Population	Phenotype	N	*kdr*		Mutant (%) frequency	OR	Genotype		
				Wild TTC(F)	Mutant TCC(S)			F/F	F/S	S/S
**Beta-cypermethrin**	YW	R	18	5	31	86.11	0.56	1	2	15
		S	6	1	11	91.67		0	1	5
	QZ	R	5	1	9	90.00	23.0*	1	0	4
		S	16	23	9	28.13		10	4	2
	WZ	R	6	5	7	58.33	0.28	1	3	2
		S	15	5	25	83.33		0	5	10
	JX	R	24	20	28	58.33	2.05	5	10	9
		S	32	38	26	40.63		11	17	4
	Total	R	53	31	75	70.75	2.28*	8	15	30
		S	69	67	71	51.44		21	27	21
**Deltamethrin**	YW	R	18	0	36	100.00	/	0	0	18
		S	16	6	26	81.25		1	4	11
	QZ	R	10	5	15	75.00	6.6*	1	3	6
		S	16	22	10	31.25		8	6	2
	WZ	R	10	5	15	75.00	1.17	0	5	5
		S	16	9	23	71.88		1	7	8
	JX	R	26	29	23	44.23	1.27	6	17	3
		S	43	53	33	38.37		15	23	5
	Total	R	64	39	89	69.53	2.23*	25	40	26
		S	91	90	92	50.55		32	65	58
**Permethrin**	YW	R	12	4	20	91.67	0.71	0	2	10
		S	16	4	28	87.5		0	4	12
	QZ	R	3	3	3	50.00	1.46	1	1	1
		S	16	19	13	40.63		7	5	4
	WZ	R	6	9	3	25.00	0.13	3	3	0
		S	14	8	20	71.43		3	4	7
	JX	R	9	7	11	61.11	3.22*	1	5	3
		S	32	43	21	32.81		12	18	2
		R	30	21	37	61.67	1.59	5	11	14
	Total	S	78	74	82	52.56		22	31	25

N indicates the sample number. S was the susceptible phenotype; R was the resistant phenotype.

*P < 0.05.

In this study, individuals with *kdr* mutations in both codons 1532 and 1534 were detected in samples obtained from both QZ and JX. The genotypes included both wild-type (I/I+F/F), wild-type+mutant (I/I+F/S, I/I+S/S, I/T+F/F), and both mutant types (I/T+F/S, I/T+S/S) (**Table 2**).

**Table 2:** Number of simultaneous mutations at codon 1532 and 1534 in *Ae*. *albopictus* populations from QZ and JX.

Population	N	(Wild+wild) type	(Wild+mutant) type			(Mutant+mutant) type	
		I/I+F/F	I/I+F/S	I/I+S/S	I/T+F/F	I/T+F/S	I/T+S/S
QZ	66	24	19	19	2	2	0
JX	166	40	85	27	10	3	1
Total	232	64	104	46	12	5	1

N indicates the sample number.

### Correlation Between Mutant *kdr* Genotypes and Resistance Phenotypes

The OR values were calculated for both mutant alleles. At codon 1532, the mutant frequency was 4.17% for susceptible individuals after exposure to beta-cypermethrin in all samples, while it was 1.72% for resistant individuals, with an OR value of 0.40 (95% CI: 0.04–3.70) (**Table 3**). The OR value was 0.54 and 0.55 in *Ae. albopitus* populations to deltamethrin and permethrin, respectively (95% CI: 0.06–5.29 and 0.07–4.72, respectively), indicating that the I1532T mutant allele might be negatively correlated with the three insecticide resistance phenotypes (**Table 3**). None of the mutant alleles at codon 1532 were found in resistant QZ individuals for all three insecticides, whereas it was found in individuals susceptible to beta-cypermethrin and permethrin (**Table 3**). No significant correlation between the I1532T mutant allele and three pyrethroids resistance phenotypes were observed, which might be caused by the relatively small sample size.

**Table 3:** *Kdr* mutant allele frequency at codon 1532 of *Ae*. *albopictus* populations in QZ and JX.

Insecticide	Population	Phenotype	N	kdr		Mutant (%)frequency	OR	95% CI	
				Wild ATC(I)	Mutant ACC(T)			Down	Up
**Beta-cypermethrin**	QZ	R	5	10	0	0.00	/	/	/
		S	16	30	2	6.25			
	JX	R	24	47	1	2.08	0.66	0.06	7.49
		S	32	62	2	3.13			
	Total	R	29	57	1	1.72	0.40	0.04	3.70
		S	48	92	4	4.17			
**Deltamethrin**	QZ	R	10	20	0	0.00	/	/	/
		S	16	32	0	0.00			
	JX	R	26	51	1	1.92	0.54	0.06	5.36
		S	43	83	3	3.49			
	Total	R	36	71	1	1.39	0.54	0.06	5.29
		S	59	115	3	2.54			
**Permethrin**	QZ	R	3	6	0	0.00	/	/	/
		S	16	30	2	6.25			
	JX	R	9	17	1	5.56	0.70	0.08	6.35
		S	32	59	5	7.81			
		R	12	23	1	4.17	0.55	0.07	4.72
	Total	S	48	89	7	7.29			

N indicates the sample number. S was the susceptible phenotype; R was the resistant phenotype.

At codon 1534, the OR values were 2.28 (P < 0.05) and 2.23 (P < 0.05) in *Ae. albopitus* populations after exposure to beta-cypermethrin and deltamethrin, respectively, indicating that the F1534S mutant allele was positively correlated with both the beta-cypermethrin and deltamethrin resistance phenotype in all samples (**Table 1**). No statistically significant differences between the F1534S genotype and permethrin resistance phenotypes were found in the all samples, but a positive correlation was observed in JX.

**Figure 3:**
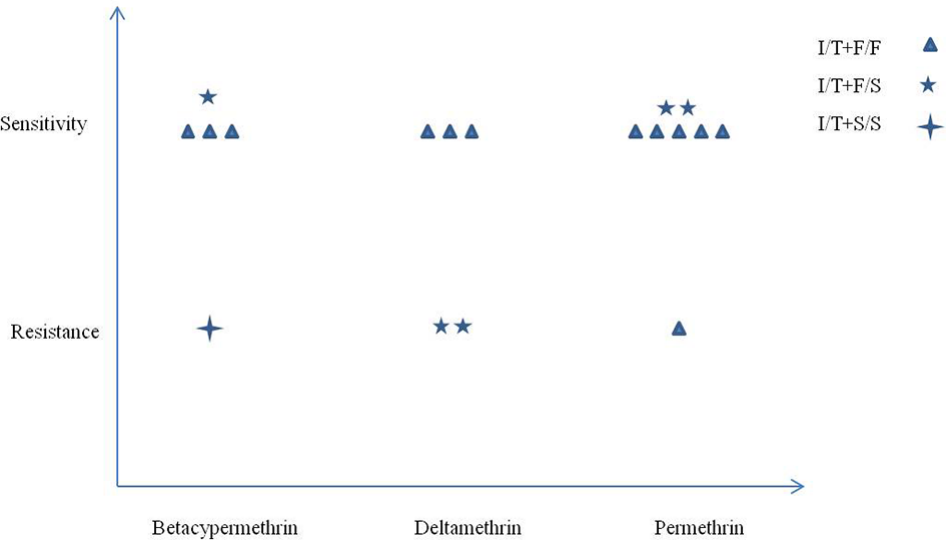
Correlation between mutations at codons 1532 and 1534 and pyrethroid resistance in *Ae. albopictus* populations from Zhejiang Province, central China.

The *kdr* mutant alleles F1534S and I1532T showed opposite effects on pyrethroid resistance. To explore the possible interactions between codons 1532 and 1534 and the resulting phenotype, all samples with the alleles I1532T+F1534, I1532T+F1534S, and I1532T+S1534 were chosen for analysis. As shown in **Figure 3**, only one sample with the I/T+S/S genotype showed resistance phenotype in three pyrethroids. A total of 83.33% (5/6) *vs.* 16.67% (1/6) of the samples with the I/T+F/F genotype were susceptible to permethrin, while almost half of the samples with I/T+F/S genotypes (2/5) had resistant phenotypes to the three pyrethroids. No statistical analysis was conducted because of the small sample size.

## Discussion

In the present study, four *Ae. albopticus* populations from Zhejiang Province in central China were tested for three pyrethroid insecticides (beta-cypermethrin, deltamethrin, and permethrin) using the WHO tube bioassay. The results showed that none of the populations were sensitive to any pyrethroid insecticide tested. After detecting the corresponding *kdr* mutations, we found mutations in domain III, specifically the I1532T mutation at codon 1532 and the F1534S mutations at codon 1534. Although the sequences of domains II and IV of VGSC were obtained, no non-synonymous mutations were found. Correlation analysis was conducted between the genotypes from codons 1532/1534 and the resistance phenotype in *Ae. albopictus*, indicating that the F1534S mutation was positively correlated with resistance phenotype to beta-cypermethrin (OR > 1, P < 0.05) and deltamethrin (OR > 1, P < 0.05), whereas the I1532T mutation was negatively correlated with resistance phenotype to beta-cypermethrin (OR < 1, P > 0.05), deltamethrin (OR < 1, P > 0.05), and permethrin (OR < 1, P > 0.05), although no statistical significance was found.

*Ae. albopictus* is responsible for transmitting various arboviruses, leading to a high health burden in Zhejiang Province, because the only regularly conducted mosquito control measures there relied mainly on chemical insecticides (Hou et al., 2017). In the urban areas of some cities in the Zhejiang Province, most of the citizens from the residential communities signed an agreement with pest control operation companies (PCO) in the quest to kill mosquitoes twice a month during the seasons of peak mosquito activity. When dengue fever outbreaks occur, the spraying frequency increases to once daily to control the spread of the disease (Gao et al., 2018). All these measures lead to the overuse of insecticides, causing resistance in mosquitoes, as well as flies, who share the same living environment. According to the data regarding house files in 2011, 2014, and 2017, resistance to pyrethroids was very common (Wang et al., 2019).

Thus, a systematic surveillance of the insecticide resistance of *Ae. albopictus* in Zhejiang Province was carried out in 2016, and it was found that the number of cities where mosquitoes were resistant to pyrethroids, organophosphorus, and carbamates increased in 2019 (National Health Commission of the People’s Republic of China, 2016; Hou et al., 2017). Compared to a previous study in 2019 in Zhejiang Province, JX and WZ showed a low mortality after exposure to all three pyrethroids, while QZ showed a low mortality after exposure to deltamethrin (National Health Commission of the People’s Republic of China, 2016). These differences might be caused by the differences in sample locations. In a previous report in 2019, to obtain the resistance level of mosquitoes in the entire city, almost half of the *Ae. albopictus* populations were collected from rural and non-human settlements where no regular spray was applied. In the present study, samples were mainly collected from residential areas and parks in the urban areas where spaying was conducted regularly (Chen et al., 2016; National Health Commission of the People’s Republic of China, 2016). Among the four populations, mosquitoes from YW showed the strongest resistance to all three pyrethroids, which was consistent with data obtained from previous reports (National Health Commission of the People’s Republic of China, 2016; Hou et al., 2017). This might be caused by a high pressure on the city regarding the handling of large numbers of dengue patients from outside the country due to the international mobility of business personnel annually. As a result, the local government had to regularly organize large-scale mosquito control campaigns using large amounts of pyrethroid insecticides (National Health Commission of the People’s Republic of China, 2016).

According to the results of this study, there were two resistance alleles, one each at codons 1532 and 1534. At codon 1534, only one mutant allele, TCC(S), was identified in mosquitoes from Zhejiang Province, central China. This was consistent with the study by Chen et al. in Shanghai and Hangzhou (both cities are from central China), but different from the results obtained from Ruili City (Yunnan Province, southern China), and Haikou City (Hainan Province, southern China), where three mutant codons, TTC(S), TGC(C), and TTG(L) were found (Chen et al., 2016; Gao et al., 2018; Lan et al., 2019). These differences should be further analyzed. We hypothesize that one influencing factor for this might be the high selection pressure on mosquitoes in Ruili and Haikou. Both cities are located in the marginal area of the tropics where *Ae.* mosquito density is high throughout the year and dengue fever outbreaks occur frequently (Li et al., 1986; Qiu and Zhao, 1988; Qiu et al., 1991; Qiu et al., 1993; Yong-hua et al., 2014; Han et al., 2015; Gao et al., 2018). Continuous use of insecticides in cities in southern China might contribute to the selection for homozygous *kdr*, where a diversity in the gene could be more conducive to the survival of the species (Brito et al., 2013). At codon 1532, the frequency of I1532T ranged from 3.03% (QZ) to 4.22% (JX). These low frequencies indicate that the mutation may have just emerged or was introduced very recently in Zhejiang Province, central China (Aguirre-Obando et al., 2017).

Few studies have reported the correlation between the *kdr* mutant allele in codon 1534 and pyrethroid resistance in *Ae. albopictus* in China. Chen et al. (2016) first identified the F1534S allele in the Haikou mosquito population and demonstrated that it was positively correlated with resistance to permethrin and beta-cypermethrin. Further, Gao et al. demonstrated that F1534S was positively correlated with resistance against permethrin and deltamethrin. In this study, our results confirmed that F1534S was significantly positively correlated with resistance against beta-cypermethrin and deltamethrin. In codon 1532, we found that the mutant allele I1532T was possibly negatively correlated with resistance against the three pyrethroids tested, with OR values ranging from 0.40 to 0.55, which were consistent with the results obtained from Shanghai City (Gao et al., 2018). However, no statistical significance was found in this study because of the relatively small sample size resulting from the low mutation frequency at codon 1532. The small sample size also made it impossible to analyze the interaction between I1532T and F1534S on the resistance phenotype. Thus, further studies with larger sample sizes are needed to study the role of I1532T in pyrethroid resistance in *Ae. albopictus*.

## Data Availability Statement

The original contributions presented in the study are included in the article/supplementary material. Further inquiries can be directed to the corresponding authors.

## Author Contributions

JS, ZG, and JH conceived the study and coordinated its implementation. JH, YyW, and QL participated in the experimental design. YQ, YpW, QN, WC, JW, TL, and ML collected mosquito samples. YyW and QL performed the experiments. JS and YyW interpreted the data. YyW and QL drafted the manuscript. All authors contributed to the article and approved the submitted version.

## Funding

This work was granted by the National Critical Project for Science and Technology on Infectious Diseases of P. R. China (no. 2017ZX10303404).

## Conflict of Interest

The authors declare that the research was conducted in the absence of any commercial or financial relationships that could be construed as a potential conflict of interest.
